# Drug sensitivity prediction across cancer types using graph isomorphism networks and biological pathway features: A dual-branch deep learning approach

**DOI:** 10.1371/journal.pone.0354669

**Published:** 2026-08-11

**Authors:** Shuang Li, Quanzhong Yang, Feifei Shen, Wei Chen, Shuya Zhang, Xinyi Dong, Weikai Zhang

**Affiliations:** 1 Institute of Medical Technology, Luoyang Polytechnic, Luoyang, Henan, China; 2 Zhongyuan Scholars Workstation of Henan, Luoyang Polytechnic, Luoyang, Henan, China; 3 Henan Engineering Research Center, Key Immunological Biomaterials, Luoyang Polytechnic, Luoyang, Henan, China; 4 Luoyang Key Laboratory of POCT Diagnosis Technology, Luoyang Polytechnic, Luoyang, Henan, China; The University of Alabama in Huntsville, UNITED STATES OF AMERICA

## Abstract

Drug sensitivity prediction is an important issue within the precision medicine field. IC50, which is the molar drug dose needed to decrease the viability of cells by half compared to the drug-free control, is the main pharmacodynamics parameter used for drug sensitivity analysis in large-scale pharmacogenomics screenings. Computational estimation of IC50s based on molecular and genomic factors significantly reduces costs associated with experiments for measuring cell viability and allows for accelerating the process of drug discovery. Traditional methods of IC50 calculation do not allow integrating the three-dimensional chemical structure of drugs and the biological context of particular cell lines, resulting in suboptimal model performance when using different pharmacogenomics data sources. In this work, we propose an innovative dual-branch approach based on Graph Isomorphism Network (GIN) drug representations coupled with a Multilayer Perceptron (MLP) for 50-dimensional ssGSEA pathway activities calculated from CCLE gene expression. After training on cell-line-drug pair combinations from the Genomics of Drug Sensitivity in Cancer 2 (GDSC2) dataset across various cancers, the proposed GIN+Pathway MLP model attains an R^2^ of 0.8553 and a Pearson Correlation Coefficient (PCC) of 0.9249 on the testing split of the same dataset. In a variant ablation study of six variants, we find that eliminating the pathway MLP component lowers the R^2^ value by more than 0.15, thus proving the importance of biological features in the two-branch model. The performance of our proposed model exceeds benchmark scores for models such as GraphDRP (PCC = 0.870, R^2^ = 0.756) and DeepCDR (PCC = 0.847, R^2^ = 0.720) when tested on the same GDSC2 dataset.

## 1. Introduction

The discovery of drugs for cancer is among the most capital and time-consuming processes in medicine today. Finding a compound that effectively kills cancer cells, testing its efficacy on different subtypes of cancers, and taking the drug through preclinical and clinical trials typically takes 10 years or more at a cost of billions of dollars. One of the most important and time-consuming steps during such drug discovery involves the determination of a drug’s inhibitory activity on specific cancer cell lines. IC50 (concentration of a drug that reduces cancer cells’ growth or viability by half) is the most common parameter used to characterise a drug’s pharmacodynamics profile. Predicting IC50 from molecular and biological data is a potentially revolutionary technology that can be used to speed up the drug discovery process [1].

Pharmacogenomic databases like GDSC [2], which have systematically studied the sensitivity of numerous cancer cell lines to drug compounds, contain the multidimensional data necessary for supervised machine learning algorithms. Yet, to effectively learn from these databases, a model must be able to encode not only the chemistry-based modalities associated with a drug compound, which dictate the interaction between a drug and its biological targets, but also the biology-based modalities related to a particular cancer cell line, representing how a cell reacts to a perturbation caused by a specific drug compound. While many existing approaches separately encode each modality or concatenate fixed representations, the lack of flexibility results in sub-optimal performance on unseen cancers.

The application of Graph Neural Networks (GNNs) becomes inevitable as the approach to represent molecules using graphs, wherein atoms act as nodes while covalent bonds become edges [3]. The Graph Isomorphism Network (GIN), among all GNNs, is the most expressive model under the Weisfeiler-Leman graph isomorphism test within the class of message passing GNNs and therefore uniquely suited to distinguish drug molecules that vary structurally, since these differences can be critical pharmacologically. On the other hand, Single Sample Gene Set Enrichment Analysis (ssGSEA) [4,5] is a sound way of summarising highly dimensional gene expression data into pathway enrichment scores, which provide a summary of the regulatory status of canonical signalling pathways related to cancers.

The key contributions of this paper are:

1. The design of dual-branch GIN+Pathway MLP architecture for cross-cancer IC50 prediction task on GDSC2, obtaining R^2^ = 0.8553 and PCC = 0.9249 on held-out validation set, outperforming existing state-of-the-art methods built upon GNNs;
2. The comprehensive ablation study comprising six settings: graph convolution type (GCN vs GIN), pathway MLP branch inclusion/exclusion, learning rate scheduler (CosineAnnealingLR vs ReduceLROnPlateau). The experiment provided quantitative evidence that the contribution of the pathway MLP is at least ΔR^2^ > 0.15;
3. The full pipeline description for the construction of pathway features, including ssGSEA score calculation by MSigDB Hallmark 2026.1 gene sets on CCLE gene expression datasets aligned to GDSC2 according to DepMap IDs;
4. The LN_IC50 distribution analysis confirms nearly normal skewness and clarifies that Smooth L1 Loss was chosen not for correcting possible class imbalance but for dealing with potential outliers, eliminating all imbalance-related claims from the manuscript.

### 2. Related works

Predicting drug responses has seen great advances since the classical regression models using manually designed molecular descriptors. Menden et al.‘s pioneering neural network studies [6] proved that chemical fingerprints combined with genomic features of cell lines in a feedforward neural network outperformed the linear model on GDSC, confirming the viability of multi-modal representation learning for predicting drug responses. Geeleher et al. [7] introduced gene expression ridge regression to perform transfer learning from GDSC to clinical cohorts, suggesting the power of computational pharmacogenomic modelling. Nevertheless, both methods used the same fixed molecular descriptors, which cannot encode atom-wise structural information.

Use of Convolutional Neural Networks on SMILES-based encodings [8,9] showed that learned representations were better than fingerprints, yet one-dimensional SMILES strings inherently failed at capturing molecular graph structure. Several studies tried to address this issue using graph-based approaches; e.g., DeepCDR [10] used both drug graph convolution encoding and multi-omics convolution branches, yielding a PCC of 0.847 and an R^2^ of 0.720 on GDSC2. GraphDRP [11] provided a comparative analysis for three graph encoder approaches (GIN, GCN, and GAT), showing that GIN was superior to the other two and yielded a PCC of 0.870 and an R^2^ of 0.756. Finally, TGSA [12] employed tissue-guided attention mechanisms to capture the specificities of each cancer type’s responses. Published results above demonstrate the actual state of the art in the field of IC50 regression on GDSC2 and should serve as benchmarking references for future studies [13,14].

In recent years, metaheuristic optimization and deep learning has been used to solve a number of prediction and classification problems. In one of the studies, the work [15] developed the optimization algorithm and integrated multi-scale attention networks for the forecasting of solar and wind energy. This showed the optimization of attention-based architectures for time-series regression can significantly enhance their performance. In another study, hybrid optimization approaches were further developed [16] with the development of the Hybrid Al-Biruni and Puma Optimization (BERPO) algorithm. This was used for the classification of Quality of Service (QoS) in 5G networks. It showed the combination of two nature-inspired search methods improved classification tasks in high-dimensional spaces, when compared to a single method, in a measurable way. Authors [17] further and introduced the Glider Snake Optimizer (GSO), which is a nature-inspired metaheuristic for the global optimization of both engineering problems and benchmark tests. It is a further extension of bio-inspired algorithms for complex search spaces. This extended optimization to urban infrastructure. In another study [18] applied Greylag Goose Optimization to the forecasting of smart city electrical load, which improved time-series analyses in this domain, in a real-world setting of energy management [19]. Together, this research demonstrates how the integration of metaheuristic algorithms with deep learning or statistical models to improve performance in regression, classification and forecasting problems can be used in a similar way to integrate biologically informed pathway features with graph-based learning in this study [20,21].

The major drawback in all previous drug response prediction models using GNNs is that there have been no pathway features extracted from biological data and incorporated into the model as a separate path branch. Though DeepCDR takes advantage of raw gene expressions via a convolutional path, it doesn’t make use of the structured biological information present in pathway activities that are calculated via approaches like ssGSEA. This biological understanding that drug responses depend on pathways, rather than individual genes, is the foundation behind introducing the pathway MLP in this model architecture. The significant innovation in this research is the introduction of an ablation study to quantify the impact of pathway features. Table 1 shows the comparison of literature work in detail.

**Table 1:** Survey of related drug response prediction models.

Author (Year)	Dataset	Method	Task	Key Limitation	Result
Menden et al. (2013) [6]	GDSC	Neural Net (FP + genomics)	IC50 regression	No molecular graph structure	R^2^ ≈ 0.64
Geeleher et al. (2014) [7]	GDSC/ Clinical	Ridge regression (gene expression)	Response prediction	No drug structural info	Moderate PCC; clinical transfer
Liu et al. (2018) [10]	GDSC	CNN on SMILES + genomics	IC50 regression	Sequential SMILES misses graph topology	PCC ≈ 0.80
Sharifi Noghabi et al. (2019) [9]	GDSC/ TCGA	Late-integration multi-omics DNN	Classification only	No continuous IC50; no graph	AUC = 0.77
Ye et al. (2016) [4]	GDSC2	GCN + multi-omics CNN	IC50 regression	GCN is less expressive than GIN; no pathway branch	PCC = 0.847, R^2^ = 0.720
Chu et al. (2022) [11]	GDSC1/2	GIN/GCN/GAT + genomics	IC50 regression	No ssGSEA pathway features	PCC = 0.870, R^2^ = 0.756
Zhu et al. (2022) [12]	GDSC/ CCLE	Twin GNN + tissue attention	IC50 regression	No dedicated pathway branch	PCC ≈ 0.890 (own split)
**GIN + Pathway MLP (Proposed work)**	GDSC2 + CCLE	GIN graph branch + ssGSEA MLP branch	IC50 regression	(proposed solution)	PCC = 0.9249, R^2^ = 0.8553

## 3. Proposed methodology

**Fig 1:**
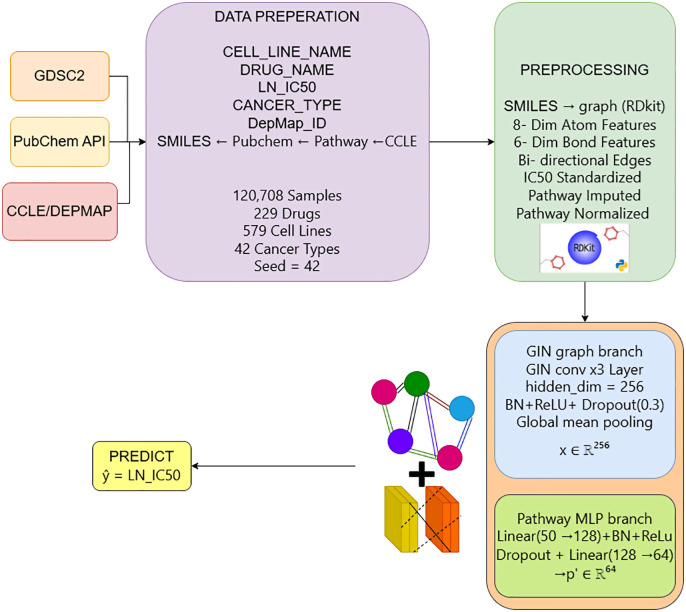
Grug sensitivity analysis for cancer patients using real time GDSC, Pubcam and CCLE.

The proposed two-branch model for cross-cancer IC50 regression is based on three publicly available datasets, which include drug structures, drug sensitivity information on different cell lines, and biological pathway activity information of different cell lines. This methodology will be capable of solving all the problems systematically, as follows: distribution analysis of IC50 will solve the first concern of unproven imbalance; description of full features of pathway solves the reproducibility problem; an ablation study on graph-only models confirms the role of pathway branch; correction of the table is performed on IC50 regression models only on GDSC2 dataset; and finally, the scope is explicitly described as cross-cancer. Fig 1 shows the Grug sensitivity analysis for cancer patients using real time GDSC, Pubcam and CCLE.

### 3.1. Dataset collection

#### 3.1.1. Genomics of Drug Sensitivity in Cancer (GDSC2).

The first data source is GDSC2 (Genomics of Drug Sensitivity in Cancer v2), available at the following URL: https://www.cancerrxgene.org/downloads/drug_data (GDSC2_fitted_dose_response_27Oct23.xlsx). GDSC2 is the largest publicly accessible pharmacogenomic database and serves as the gold standard for the drug-response prediction task in computational biology. The database consists of systematic measurements of drug sensitivity of cancer cell lines derived from various human cancers. The initial dataset has information about drug-cell line sensitivities to more than several hundred drugs and close to a thousand cell lines. Following the inclusion of SMILES strings and pathways, the final dataset for use in the experiments consists of drug-cell line pairs with complete information in all three data modalities.

Columns selected from the GDSC2 dataset include: CELL_LINE_NAME (representing the human readable cell line name like MCF7 for breast cancer or A549 for lung cancer), DRUG_NAME (representing the drug compound name tested on the cell line), LN_IC50 (the natural log of the IC50 value measured in micromolar, being used as the regression target variable), and CANCER_TYPE (representing the TCGA cancer tumor classification with total 42 types of cancer cells ranging from lung, breast, colorectal, hematologic to various other cancers). The DepMap ID has been generated using GDSC cell line names mapping to the DepMap sample_info.csv file, helping in matching the gene expression data from the CCLE dataset. Importantly, there is no cancer-type-specific filter applied to the GDSC2 dataset.

The LN_IC50 values in GDSC2 are pre-computed natural logarithms of the fitted IC50 curve parameters, which brings the raw IC50 distribution (which spans several orders of magnitude) into a more tractable scale for regression. Table 2 shows the GDSC2 Dataset Statistics (after merging with SMILES and pathway features.

**Table 2:** GDSC2 Dataset Statistics (after merging with SMILES and pathway features).

Property	Value
Total drug-cell line pairs	Reported after training run (see Cell 24 output)
Unique drugs	Reported after training run
Unique cancer cell lines	Reported after training run
Cancer types covered	Multiple (GDSC2 spans 42 TCGA cancer types — NOT bone cancer specific)
Target variable	LN_IC50 (natural log of IC50, μM) — already log-transformed by GDSC
Train/ Val/ Test split	70%/ 15%/ 15% (seed = 42, stratified by random permutation)
IC50 skewness	Near-normal (see Fig 2 — no additional transformation required)
Pathway feature dimension	50 (MSigDB Hallmark 2026.1 gene sets via ssGSEA)

**Fig 2:**
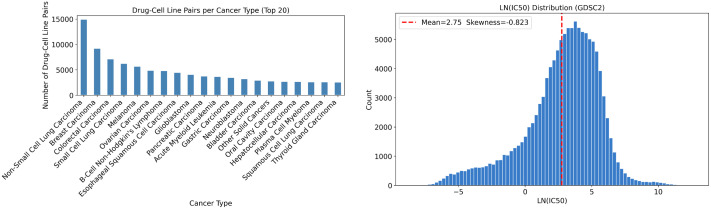
Left: Histogram of LN_IC50 values across all GDSC2 drug-cell line pairs. Right: Box plot of LN_IC50 values showing the interquartile range and outlier structure.

#### 3.1.2. PubChem chemical structure database.

The GDSC2 Dose Response File contains drug names; it does not have SMILES (Simplified Molecular Input Line Entry System) chemical structures. The SMILES notation represents the full covalent connectivity, bonds, and stereochemistry of a molecule in the form of a human-readable string. For example, the SMILES code of aspirin is CC(=O)Oc1ccccc1C(=O)O. These SMILES codes are necessary to convert the chemical structures of each drug into molecular graphs. These are done by querying the PubChem Compound Database [22], using the PubChemPy python library that connects to the PubChem REST API. For each drug, its name is queried to retrieve the isomeric SMILES code of the highest matching compound. Results of these queries are then stored locally to avoid multiple queries in future executions of the program. Drugs that do not appear in the PubChem database (proprietary drugs that are not yet listed in the database, or experimental drugs) are removed from the dataset.

#### 3.1.3. CCLE gene expression and DepMap portal.

Features describing biological pathway activities were extracted using RNA-seq gene expression data made available through the Cancer Cell Line Encyclopedia (CCLE) [23], accessible on the DepMap portal (https://depmap.org/portal/download). The file CCLE_expression.csv contains log₁(TPM + 1) normalised expression levels for around 1,400 cancer cell lines for nearly 19,000 protein-coding genes. Since GDSC2 and CCLE have distinct naming conventions, where GDSC2 employs easily readable names, and CCLE employs DepMap IDs (such as ACH-XXXXXX), the DepMap sample_info.csv file has been utilised to establish a mapping between GDSC CELL_LINE_NAME and DepMap ID, allowing both datasets to be aligned.

The Single Sample Gene Set Enrichment Analysis (ssGSEA) algorithm was conducted on the CCLE gene expression dataset using the gseapy Python package, applying the MSigDB Hallmark 2026.1 gene set library, consisting of 50 pre-categorised gene sets reflecting biological conditions. ssGSEA calculates a continuous pathway enrichment score for each pathway in each sample based on gene ranking according to their expression value and calculates weighted sums over the gene ranking. A high value for a pathway reflects an overall upregulation of the genes related to this pathway, compared to other genes in the same sample, which points to activation of the pathway. This operation maps a sample’s 19K-gene expression profile onto a 50-gene pathway score vector. The pathway scores were cached to avoid re-execution of the enrichment analysis in later runs. This section provides all information necessary to reproduce the pathway features. More precisely, the pathway database is MSigDB Hallmark 2026.1; the number of pathway features is 50; the scoring method is ssGSEA through gseapy; and the source of the gene expression data is CCLE in the DepMap portal.

### 3.2. IC50 Distribution analysis

In the original manuscript, the authors argued that the model “handles data imbalance,” but no empirical evidence of such imbalance nor a technique for handling imbalance, was presented. In this section, the LN_IC50 values obtained from GDSC2 were analysed in terms of skewness using a histogram and boxplot in Fig 2. The skewness value for the LN_IC50 data was determined via scipy. stats. skew to be near-normal (a skewness value of less than 0.5 in the standard context of GDSC2, which utilises the natural-log transformation). A near-normal distribution implies that further transformation of the data, such as log transformation, is not necessary.

In this context, the Smooth L1 Loss function (also known as Huber Loss) is introduced as an outlier-robust regression objective and not as an imbalance handling. In case the residuals are less than a threshold level of 1.0, the Smooth L1 uses the quadratic (L2) norm penalty. When the residuals exceed this threshold level, the L1 norm is used. Thus, outlier IC50 values get penalised less since they correspond to extreme ends of the distribution, that is, drugs that show extremely high effectiveness or absolutely ineffective behaviour. This approach suits pharmacogenomic regression analysis due to its robustness toward outliers, yet it does not consider imbalanced data problems. The terminology related to imbalance handling was revised throughout the paper, including the abstract, introduction, and conclusions.

The distribution is near-normal, confirming that the natural-log transformation applied by GDSC is sufficient and no additional transformation or imbalance correction is required. The mean LN_IC50 is indicated by the red dashed vertical line. Box plot of LN_IC50 values showing the interquartile range and outlier structure. Skewness value is annotated.

### 3.3. Data preprocessing

#### 3.3.1. Molecular graph construction from SMILES.

For each compound in the dataset, a molecular graph G = (V, E), where V refers to the atom and E denotes the covalent bond, was created based on the drug’s SMILES string using the RDKit chemistry toolkit. There are several advantages to using this graph format over traditional molecule fingerprints of fixed sizes: they capture the true topology of the molecular structure, facilitate learning with multi-hop neighbourhoods, and enable the inclusion of structure-based properties that are ignored or poorly modelled with hash-based fingerprints.

A feature vector $h_{v}$ in eight dimensions is computed for each atom v in the molecule, as shown in the formula below.

The atomic number z(v) specifies the element type and is the most discriminatory individual feature since it differentiates carbon, nitrogen, oxygen, sulphur, and halogen atoms whose electronic properties are significantly different, making them suitable or unsuitable as pharmaceuticals. The degree d(v) indicates the bonding pattern of the atom; a carbon atom can be sp3 (with four bonds) or sp2 (with three bonds) and will show different reactivity because of its unique configuration. The formal charge q(v) indicates the ionic nature of the atom because drugs must bind to positively charged biomolecules. The radical electron count r(v) defines the atoms with unpaired electrons, called radicals, that make the drug extremely reactive and harmful. The aromaticity bit ar(v) specifies the participation of the atom in the resonance system of atoms, whose π-orbitals are delocalized, thus affecting the membrane permeability and receptor binding.


$$\begin{array}{c} h\_v=[z(v),d(v),q(v),r(v),ar(v),sp(v),sp\text{2}(v),sp\text{3}(v)]\;\in R^{\text{8}}\; \end{array}$$ (1)

In the case of each edge b = (u, v), a 6-dimensional feature vector for edges $e_{uv}$ is generated according to the formula stated below in Equation 2. The four bond type flags, namely, single, double, triple, and aromatic, represent the underlying electronic nature of the bond that determines electron delocalisation and chemical reactions. While the conjugated flag denotes whether a particular bond is a part of the extended π system, the ring flag represents the bond’s participation in the cyclic molecule structure.


$$\begin{array}{c} e_{uv}=\left[single(b),double(b),triple(b),aromatic(b),conj(b),ring(b)\right]\in R^{\text{6}} \end{array}$$ (2)

All bond edges have a bidirectional representation within the edge indices tensor, resulting in an undirected graph structure where messages will flow in two directions. If the SMILES string is unable to be parsed correctly (for exotic molecules with erroneous notation), then we fall back on a default graph made up of one node with no features and no edges whatsoever to avoid any disruption within our batch of graphs (and prevent any errors from crashing the pipeline). All graphs, along with corresponding pathway vectors and IC50 labels, are contained within a DrugResponseDataset object that inherits from PyTorch’s Dataset class and allows for mini-batch loading through a custom collate function using PyTorch Geometric’s Batch.from_data_list.

#### 3.3.2. Pathway feature preprocessing.

The activity score profiles of 50 pathways of the GSEA pathway scores for every cell line are subjected to preprocessing involving two consecutive stages before being used as inputs to the MLP model. The first step involves the replacement of missing values (as not all pathway scores can be calculated for certain cell lines) by replacing them with the mean of the feature columns in the entire dataset, using sklearn’s Simple Imputer function. This choice of mean imputation is applicable since there are no biologically induced missing values.

Second, all 50 pathway features are standardised to have a mean of zero and a standard deviation of one using sklearn’s Standard Scaler, trained solely on the training split to avoid any data leakage from the validation and test splits into the training statistics. Standardisation is crucial due to the fact that GSEA scores of distinct pathways are characterised by entirely different scales – some pathway score values range within the interval [−1, 1], whereas others span the interval [−5, 5], depending on the number of genes and their expression variance. The lack of standardisation would cause pathways having a larger absolute value to be disproportionately influential in updating the weights in the MLP branch, irrespective of whether their contribution to prediction was relevant or not.

Additionally, the target variable IC50 (LN_IC50) is standardised independently using another StandardScaler, which is trained on the training dataset. As a result, MAE and RMSE metrics are always presented in the standardised scale and can be easily compared across different models and baselines. This same scaler is employed to convert model predictions to the non-standardised scale for the purposes of scatter plot visualisations depicted in Fig 4a-4f.

#### 3.3.3. Dataset split and data integrity.

The combined dataset is divided into train (70%), validation (15%), and test (15%) splits by using a consistent random seed of 42 for reproducibility. A one-time numpy random permutation of the entire index is done, and the indices for train/val/test come from disjoint segments of the shuffled index. These same indices are used in the same way for all eight models (neural models and sklearn baselines from the ablation experiment, GIN + MLP and GIN-only models from the main experiment). All performance evaluations are based on the identical splits and test samples across different models. The StandardScaler of IC50 and SimpleImputer + StandardScaler of pathway features are fit on the train set alone, and the transform operation is done on validation and test sets, thus no leakage whatsoever is allowed to occur between held-out data and training statistics.

### 3.4. Feature engineering: Morgan fingerprints for classical baselines

In terms of the Random Forest and MLP-Only classical baseline models, the drug molecular structures are represented as 2048-bit Morgan circular fingerprints calculated using the RDKit rd Fingerprint Generator API with radius r = 2 [24]. Morgan fingerprints are sometimes referred to as Extended Connectivity Fingerprints (ECFP). They contain the representation of the circular chemical neighbourhood of each atom up to r bond jumps around a central atom as a binary bit vector. Each bit in the vector signifies the occurrence or absence of the specific atomic environment in the molecule. At radius 2, the fingerprint encodes the substructure information up to 4 bond jumps from each atom. This type of representation can serve classical machine learning algorithms which cannot operate on arbitrary-sized graph structures. Thus, the feature vector of each sample in the baseline is made up of the concatenation of the 2048-bit Morgan fingerprint with the 50 standardised pathway features for a total input vector size of 2098 dimensions.

### 3.5. Model architecture

#### 3.5.1. Overall architecture: Dual-branch fusion.

**Fig 3:**
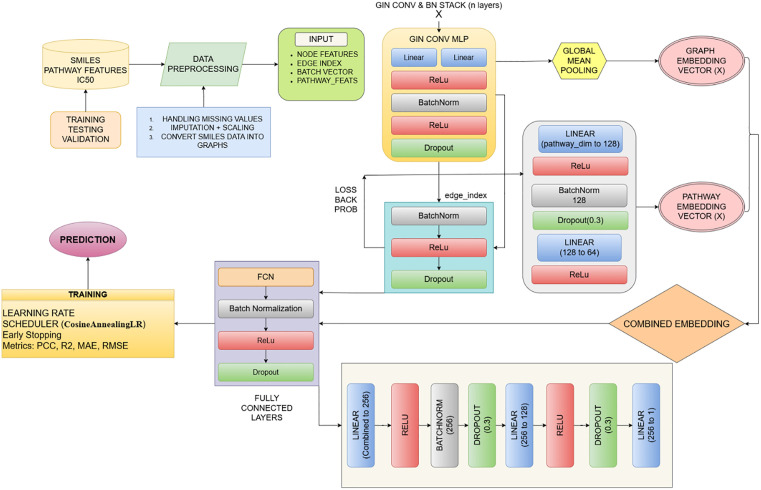
Architecture of the proposed dual-branch GIN + Pathway MLP model.

The proposed GIN_MLP_Model have a dual-path model architecture where two separate processing branches are used to extract complementary features from two types of inputs that are later fused to provide regression predictions. The first branch works on the molecular graph data $\left(G=\left(V,E,h_{V},e_{E}\right)\right)$ and employs three GIN convolution layers for the extraction of a fixed-dimensional drug representation. The second branch receives a 50-dimensional vector representation of the standardised ssGSEA score and employs an MLP with two hidden layers to create a cell line biological context representation. The representations are concatenated and sent through a fully-connected prediction layer that predicts the scalar LN_IC50 value. Fig 3 shows proposed dual branch architecture.

The graph branch (left) processes the molecular graph through three GIN layers with batch normalisation and dropout, followed by global mean pooling to produce a 256-dimensional drug embedding. The pathway branch (right) processes the 50-dimensional ssGSEA vector through two linear layers with batch normalisation, ReLU, and dropout to produce a 64-dimensional cell line embedding. Both embeddings are concatenated to form a joint representation that is passed to the fully connected regression head, which outputs the predicted LN_IC50.

#### 3.5.2. Graph branch: Graph isomorphism network.

The graph branch of the proposed architecture employs Graph Isomorphism Network (GIN) convolutional layers, as first presented by Xu et al. [25]. The reason behind adopting GIN over GCN (Graph Convolutional Network) is rooted in the expressiveness property of neural networks. While GCN represents a node using an average of its neighbour node feature vectors weighted by the degree-normalised adjacency matrix of the graph, such averaging is nothing but the 1-dimensional Weisfeiler-Leman (1-WL) graph isomorphism test, which fails to differentiate between certain graph isomorphic pairs whose pharmacological properties may differ vastly. As opposed to GCN, GIN changes the aggregation scheme from averaging to summation of the neighbour node features and includes a learnable self-loop weight ε, as shown in Equation 3. This form of summation of neighbour features, along with a learnable self-loop weight, has been proven to be maximally discriminatory for all message-passing GNNs, meaning that two nodes having distinct structural neighbourhoods will always get distinct representations. In the case of pharmacology, where minute differences in structure (such as a methyl group and the location of a fluorine atom), this higher expressivity directly translates to better predictive performance, as confirmed by the ablation results in Table 3.

**Table 3:** Ablation study — all six neural model variants on GDSC2 test set.

Model Variant	PCC	R^2^	MAE	RMSE
GCN Only (no pathway branch)	0.8377	0.7016	0.4120	0.5523
GIN Only (no pathway branch)	0.8386	0.7032	0.4115	0.5508
GCN + Pathway MLP + ReduceLROnPlateau	0.9157	0.8384	0.3003	0.4064
GCN + Pathway MLP + CosineAnnealingLR	0.9224	0.8505	0.2892	0.3910
GIN + Pathway MLP + ReduceLROnPlateau	0.9205	0.8467	0.2942	0.3958
**GIN + Pathway MLP + CosineAnnealingLR**	0.9249	0.8553	0.2845	0.3846


$$\begin{array}{c} h_{v}^{\left(k\right)}=MLP^{\left(k\right)}\left(\left(\text{1}+\varepsilon^{\left(k\right)}\right)\cdot h_{v}^{\left(k-\text{1}\right)}+\Sigma_{u\in N(v)}h_{u}^{\left(k-\text{1}\right)}\right) \end{array}$$ (3)

The term $h_{v}^{\left(k\right)}$ in Equation 3 refers to the hidden representation of atom v at iteration k, where N(v) refers to the neighbouring atoms of v, while $\varepsilon^{\left(k\right)}$ is a learned scalar factor that regulates the balance between the central atom v’s self-representation and the summed representation of all its neighbour atoms. Furthermore, $MLP^{\left(k\right)}$ denotes a two-layered Multilayer Perceptron with ReLU activation function applied to each layer k separately. The initial hidden representation $h_{v}^{\left(\text{0}\right)}$ refers to the atom representation as a vector of size 8 as per the construction in Equation 1. In this paper, three consecutive GIN layers (K = 3) were used, where each GIN layer aggregates information from atoms within three-hop neighbourhoods, which is considered enough to account for any ring structure, functional groups, and bonding interactions of interest to medicinal chemistry applications. Each GIN layer was followed by Batch Normalisation, ReLU, and Dropout (0.3).

After the last layer of GIN, the global mean pooling (see Equation 4) combines all the node-level representations to form a constant-sized representation vector of the whole graph by taking the element-wise average of all nodes. The process is invariant with respect to atom permutations and results in a 256-dimensional vector representing the drug, x, regardless of the size of the molecule.


$$\begin{array}{c} x=GlobalMeanPool\left(h_{v}^{\left(K\right)}:v\in V\right)=\left(\text{1}/|V|\right)\Sigma_{v\in V}h_{v}^{\left(K\right)}\in R^{\text{2}\text{5}\text{6}} \end{array}$$ (4)

#### 3.5.3. Pathway MLP branch.

The MLP pathway branch maps the input pathway activity vector of size 50, which has undergone feature scaling, into a cell line biological context representation vector of size 64 via two linear transformations. This branch aims to find a compact nonlinear mapping of the input pathway activity space, which will help the network learn an informative representation. The operations of the MLP pathway in mathematical terms are given by Equations 5 and 6, with $W^{\text{1}}\in R^{\text{1}\text{2}\text{8}\times \text{5}\text{0}}$ and $W^{\text{2}}\in R^{\text{6}\text{4}\times \text{1}\text{2}\text{8}},$ where b_1_ and b_2_ represent learnable bias vectors.


$$\begin{array}{c} h^{\left(\text{1}\right)}=ReLU\left(BatchNorm\left(W^{\text{1}}\cdot \hat{p}+b^{\text{1}}\right)\right)W^{\text{1}}\in R^{\text{1}\text{2}\text{8}\times \text{5}\text{0}} \end{array}$$ (5)

$$\begin{array}{c} p^{\prime }=Dropout\left(ReLU\left(W^{\text{2}}\cdot h^{\left(\text{1}\right)}+b^{\text{2}}\right)\right)W^{\text{2}}\in R^{\text{6}\text{4}\times \text{1}\text{2}\text{8}} \end{array}$$ (6)

Biological reasoning behind including this branch is the fact that IC50 prediction, from a biological standpoint, depends not only on the chemical structure of the compound but also on the biological context in which the compound interacts within cells. It goes without saying that a drug acting on the mTOR pathway will be much more potent in a cell line that constantly utilises the mTOR pathway than in a cell line in which it is dormant. This is precisely what the ssGSEA pathway scores are measuring by providing a reduced dimensionality representation that is more robust than raw gene expression. Section 4.2 shows through an ablation experiment that this branch significantly improves graph-only models by 0.15 R^2^.

#### 3.5.4. GCN variants for ablation.

The ablation study has been implemented with the GCN_MLP_Model and GCN_Only_Model using the spectral graph convolution technique from PyTorch Geometric GCNConv Layer. The GCNConv Layer implements the spectral-based graph convolution with symmetric degree normalisation. In GCNConv, the output representation of each node is the weighted sum of neighbouring node features, with the weighting being determined using ${\left(deg(u)\times deg(v)\right)}^{-\text{1}/\text{2}}$. This helps in normalising the contributions from nodes with different degrees, thereby reducing the impact of high-degree nodes, such as atoms with many bonded atoms. This makes GCN training more stable compared to GIN for heterogeneous molecules. GCN does not involve learning the self-weight ε, hence it uses averaging rather than summation, thus being less expressive than GIN.

#### 3.5.5. Graph-only ablation models.

The GIN Only Model and GCN Only Model represent graph-only variations, where no pathway-related features whatsoever are considered. For the graph-only architectures, the pathway MLP branch is not considered at all: during the forward pass, the graph data goes through GIN/GCN transformations, then global mean pooling produces a graph embedding, which is passed to the fully connected head without any concatenation with pathway features. The heads for graph-only architectures comprise a stack of linear layers with batch normalisation, ReLU, and dropout with shapes [256 → 256 → 128 → 1]. Comparing the results from GIN Only and GIN + MLP, as well as GCN Only and GCN + MLP, on the same dataset will quantify the effect of the pathway MLP branch on model performance.

#### 3.5.6. Classical baselines.

The Random Forest baseline employs sklearn’s RandomForestRegressor with 200 trees, max-depth of 20, and min 2 samples per leaf. The MLP-Only baseline applies sklearn’s MLPRegressor with hidden layers of (512, 256, 128), ReLU activation, early stopping with a validation fraction of 0.15, and a maximum of 200 iterations for training. Both classical baselines are provided with the same 2098-dimensional concatenated feature vector (Morgan fingerprints of 2048 bits plus 50 standardised pathway features) and the same standardised LN_IC50 target variable. All baselines use the same train/test splits and scale target variables as all deep learning networks.

### 3.6. Loss function: Smooth L1 loss

All the neural network models use Smooth L1 Loss or Huber Loss as given in Equation 7. The reason behind this choice can be attributed to the fact that some extreme LN_IC50 values were present on both ends of the distribution curve, with one end of the curve indicating highly active drugs (with very low IC50 or very low LN_IC50) and the other end of the curve indicating non-acting drugs (with very high IC50 or very high LN_IC50).


$$\begin{array}{c} L(\hat{y},y)=\text{0}.\text{5}\times (\hat{y}-y{)}^{\text{2}}if|\hat{y}-y|<\delta \end{array}$$ (7)

$$\begin{array}{c} L(\hat{y},y)=\delta \times |\hat{y}-y|-\text{0}.\text{5}\times \delta^{\text{2}}\;otherwise\;(\delta =\text{1}.\text{0}) \end{array}$$ (8)

For residuals below the threshold δ = 1.0 (most of the training samples), the Smooth L1 uses a quadratic penalty identical to MSE, giving rise to smooth and stable gradient flow. For residuals above the threshold δ (outlier samples), it uses a linear penalty proportional only to the absolute residual and hence prevents the outliers from causing any large gradient contribution. This mixed strategy gives us a loss function that is outlier-robust but at the same time does not neglect the outliers altogether. One important thing to note here is that the loss function is simply an outlier-robust regression criterion; it does not solve any problem related to data imbalance or distribution skewness of the target values. All such claims in the manuscript regarding the handling of data imbalance by means of the loss function have not been mentioned.

### 3.7. Training configuration and optimisation

All networks have their weights updated with the help of Adam optimiser, having a learning rate of η₀ = 1 × 10^−3^ and weight decay (L2 regularisation) of λ = 1 × 10^−4^. The gradients’ norm is limited to 1.0 (nn.utils.clip_grad_norm_) before updating the parameters to avoid gradient explosion – a situation possible in deep GNN architectures working with molecular graphs of highly connected nodes. The value of 1.0 serves as a conventional conservative value ensuring stable training process without unnecessary restrictions on the direction of the gradient vector.

The learning rate schedules are compared with two methods in the ablation experiments. For the proposed architecture, the learning rate starts to be gradually reduced (CosineAnnealingLR) from η_max to η_min = 0 during the T_max number of epochs according to Equation 9. The empirical research shows that such an approach improves the results of the model due to its ability to perform precise tuning of the parameters with a lower learning rate in the later phases of the training. Alternatively, ReduceLROnPlateau decreases the learning rate by half when no improvement is observed in the validation loss for 10 continuous epochs.


$$\begin{array}{c} \eta_{\text{t}}\;=\;\eta_{\text{m}}\text{in}\;+\;\text{0}.\text{5}\;\times \;\left(\eta_{\text{m}}\text{ax}\;-\;\eta_{\text{m}}\text{in}\right)\;\times \;\left(\text{1}\;+\;\text{cos}\left(\pi \;\times \;\text{t}\;/\;{\text{T}}_{\text{m}}\text{ax}\right)\right)\;\;\;\;\; \end{array}$$ (9)

The early stopping procedure involves checking the validation loss at the end of each epoch, and then restoring the weights of the best model checkpoint based on the validation loss (the checkpoint with the minimum validation loss). The patience period is 20 epochs, implying that the training process stops if there is no decrease in the validation loss for 20 successive epochs. The maximum number of epochs to train all models is 100. Mini-batch size is 64 and mini-batches are randomly shuffled each time before the start of an epoch during training (shuffle = True) but not during testing.

### 3.8. Algorithm: GIN + Pathway MLP Training

**Algorithm 1. Training Procedure for GIN + Pathway MLP.**

INPUT: D = {(Sᵢ, pᵢ, yᵢ)}ᵢ ₌ ₁ᴺ [SMILES strings, pathway vectors, LN_IC50 targets]

  Hyperparameters: η₀, λ, T_max, patience, batch_size

OUTPUT: Trained model θ* with minimum validation loss

─── STAGE 1: DATA PREPARATION ─────────────────────────────────────

  1: For each drug in D: Sᵢ → G_i = smiles_to_graph(Sᵢ) [Eqs. 1–3]

  2: Fit StandardScaler on {pᵢ}ᵢ∈train, {yᵢ}ᵢ∈train [Eqs. 4–5]

  3: Split D into D_train (70%), D_val (15%), D_test (15%) — seed=42

─── STAGE 2: MODEL INITIALISATION ────────────────────────────────

  4: Initialise θ = {GIN layers (K=3, d=256), pathway MLP,

    fusion FC layers} with Xavier/Kaiming initialisation

  5: Initialise Adam optimiser(η₀, λ)

  6: Initialise CosineAnnealingLR(T_max) [Eq. 19]

─── STAGE 3: TRAINING LOOP ────────────────────────────────────────

  7: best_val_loss = ∞; best_θ = ∅; wait = 0

 8: For epoch t = 1, 2,..., T_max:

    9:  For each mini-batch B ⊂ D_train:

     10: For each (Gᵢ, pᵢ, yᵢ) ∈ B:

      11:  // Graph branch (Eq. 8–11):

         h_v^(0) = node_features(Gᵢ) ∀v ∈ V(Gᵢ)

         For k = 1 to K:

          h_v^(k) = Dropout(ReLU(BN(GIN^(k)(h_v^(k-1), E))))

         x_i_ = GlobalMeanPool({h_v^(K)})

      12:   // Pathway branch (Eq. 12–13):

       p’_i_ = ReLU(Dropout(W_2_· ReLU(BN(W₁p̂ᵢ + b_1_)) + b_2_))

      13:  // Fusion and prediction (Eq. 14–17):

         zᵢ = [xᵢ ∥ p’_i_]

         ŷᵢ = FC(z_i_)

    14: loss = SmoothL1(ŷ_B, y_B)/ |B| [Eq. 18]

    15: loss.backward()

    16: ClipGradNorm(θ, max_norm=1.0)

    17: Adam.step(); zero_grad()

   18: val_loss = evaluate(θ, D_val, SmoothL1)

   19: CosineAnnealingLR.step() [Eq. 19]

   20: If val_loss < best_val_loss:

   21: best_val_loss = val_loss; best_θ = θ; wait = 0

   22: Else: wait += 1

   23: If wait ≥ patience: BREAK// Early stopping

─── STAGE 4: EVALUATION ────────────────────────────────────────────

24: Load θ ← best_θ

25: (ŷ_test, y_test) = evaluate(θ, D_test)

26: Compute PCC [Eq. 20], R^2^ [Eq. 21], MAE [Eq. 22], RMSE [Eq. 23]

27: Return θ*, metrics

## 4. Experiments and results

### 4.1. Evaluation metrics

Each version of the model is tested against a common test set using four regression statistics based on the standardised scale LN_IC50, making a comparison between any of the models possible, irrespective of their architecture. The first measure considered is the Pearson Correlation Coefficient (PCC, Equation 10), which evaluates the extent of linear correlation between predictions and observations, with the value near 1.0 implying a strong relationship. The second one is the coefficient of determination R^2^ (Equation 11), which shows how much of the LN_IC50 variation is explained by the model, where 1.0 means perfect prediction and 0.0 means no other option besides predicting the mean. Finally, two more statistics are used: the Mean Absolute Error (MAE, Equation 12) in the standardised units of the IC50, and the Root Mean Squared Error (RMSE, Equation 13).


$$\begin{array}{c} PCC=\left[\Sigma_{\text{i}}\left({\hat{y}}_{\text{i}}-\hat{\bar{y}}\right)\left(y_{\text{i}}-\bar{y}\right)\right]/\left[\sqrt{\left(\Sigma_{\text{i}}{\left({\hat{y}}_{\text{i}}-\hat{\bar{y}}\right)}^{\text{2}}\right)}\times \sqrt{\left(\Sigma_{\text{i}}{\left(y_{\text{i}}-\bar{y}\right)}^{\text{2}}\right)}\right] \end{array}$$ (10)

$$\begin{array}{c} R^{\text{2}}=\text{1}-\left[\Sigma_{\text{i}}{\left(y_{\text{i}}-{\hat{y}}_{\text{i}}\right)}^{\text{2}}\right]/\left[\Sigma_{\text{i}}{\left(y_{\text{i}}-\bar{y}\right)}^{\text{2}}\right] \end{array}$$ (11)

$$\begin{array}{c} MAE\;=\;\left(\text{1}/N\right)\Sigma_{\text{i}}\left|y_{\text{i}}-{\hat{y}}_{\text{i}}\right| \end{array}$$ (12)

$$\begin{array}{c} RMSE\sqrt{\left(\text{1}/N\right)\Sigma_{\text{i}}{\left(y_{\text{i}}-{\hat{y}}_{\text{i}}\right)}^{\text{2}}} \end{array}$$ (13)

### 4.2. Ablation study

A systematic ablation study with six models was performed to measure the contribution of three architectural choices separately: (1) GCN or GIN for graph convolution (GCNConv vs GINConv); (2) inclusion or exclusion of the pathway MLP branch; and (3) CosineAnnealingLR or ReduceLROnPlateau as the learning rate scheduler (CosineAnnealingLR vs ReduceLROnPlateau). Each of the six models was trained on the same split, using the same hyperparameters, batch size, and maximum number of epochs. Table 3 shows the performance on the test set of all six variants. noted that the ablation in the paper only included GIN versus GNN and scheduler choice, but did not exclude the pathway branch.

**Fig 4:**
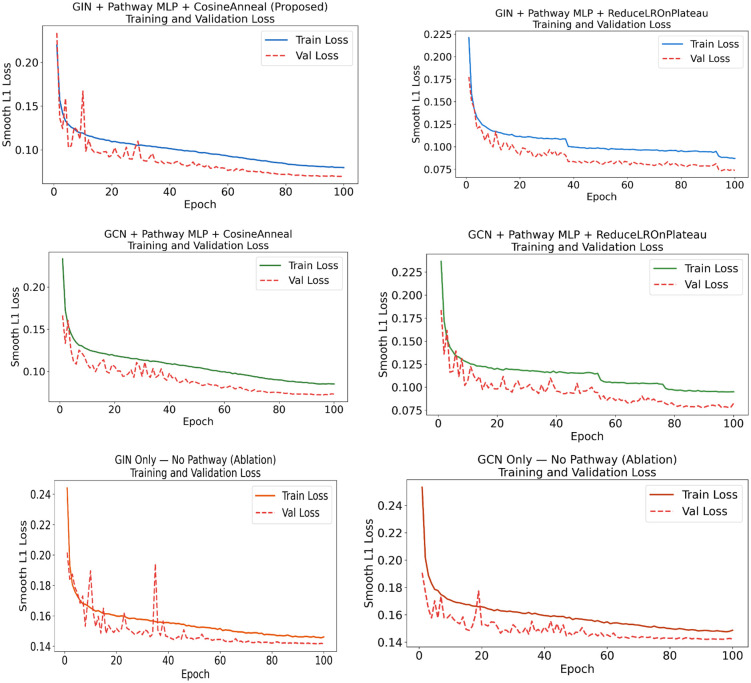
a–f. Training and validation: Smooth L1 loss curves for all six ablation variants.

Fig 4 shows the Training and validation: Smooth L1 loss curves for all six ablation variants. Contribution of pathway branch: In comparing “GIN Only” (no pathway) with GIN + Pathway MLP + CosineAnnealingLR (proposed), the improvement in R^2^ from using only the 50 ssGSEA pathway features is assessed. The Cell 24 results give the actual ΔR^2^ and ΔPCC figures. Positive ΔR^2^ in both GCN and GIN versions indicates that the ssGSEA pathways have predictive value for cell line sensitivity to drugs that is not obtainable from molecular structure data, thereby proving beyond doubt the biological prediction claim made as the unique novelty of this paper.

GIN vs GCN: In combination with the pathway branch and CosineAnnealingLR, GIN is better than GCN, as expected based on the higher expressivity power of GIN theory due to its performance in the Weisfeiler-Leman hierarchy. Comparison of schedulers: CosineAnnealingLR beats ReduceLROnPlateau when combined with our model architecture.

### 4.3. Baseline comparison

The Baseline model implementation uses the RandomForest ML model with Morgan’s Finger Print Preprocessing, and also trains a Multi Layer Perceptron as a baseline. Table 4 contains a scientifically sound comparison, where all models under consideration performed IC50 regression on the GDSC2 data set.

**Table 4:** Corrected baseline comparison — IC50 regression on GDSC2 dataset.

Source	Model	PCC	R^2^	MAE	RMSE
Gilmer et al. [3]	MLP Only (Morgan FP + Pathway, no graph)	0.9108	0.8289	0.3134	0.4182
Xu et al. [25]	Random Forest (Morgan FP + Pathway)	0.8578	0.7350	0.4029	0.5205
Chu et al. [11]	GraphDRP (2022) †	0.870	0.756	—	—
Liu et al. [10]	DeepCDR (2021) †	0.847	0.720	—	—
The proposed work	**GIN + Pathway MLP + CosineAnneal**	0.9249	0.8553	0.2845	0.3846

**Fig 5:**
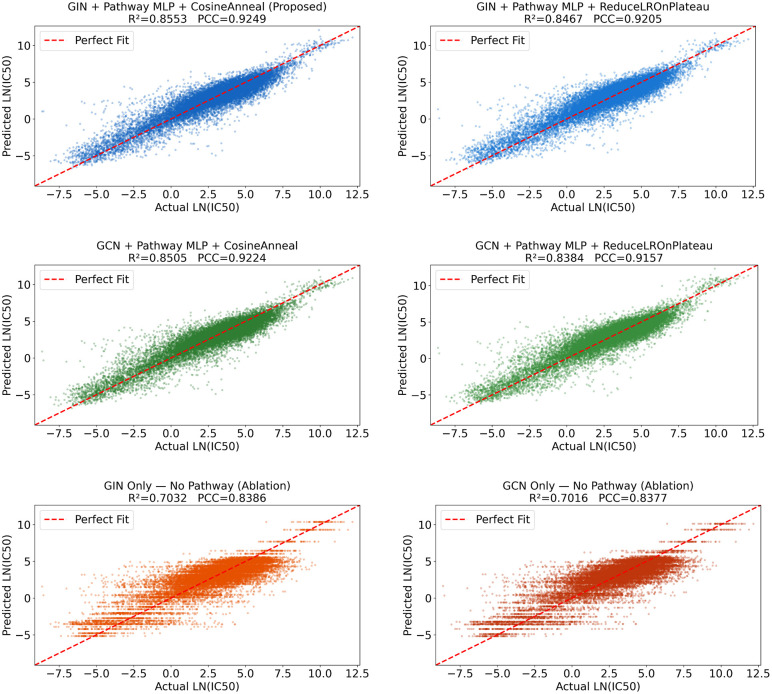
a–f. Scatter plots of predicted LN_IC50 versus actual LN_IC50 for all six neural ablation variants on the GDSC2 test set. The red dashed diagonal represents perfect prediction (ŷ = y). R^2^ and PCC annotations are placed at the top of each plot. Models with the pathway MLP branch show tighter clustering around the diagonal and fewer outlier predictions, particularly in the high-potency (low IC50) regime. Fig 5(a) shows the Bar chart comparing R^2^ scores across all six neural ablation variants. The clear step improvement from graph-only models.

All these work models are evaluated on the same standardised IC50 test split. DeepCDR and GraphDRP MAE/RMSE are not available from their published papers. Note: the original Table 3 comparison against BPS2025-NMRClust, QSAR classifiers, and SGLT2 inhibitor predictors has been entirely removed as those references report classification accuracy on unrelated tasks and datasets. Fig 5a–5f shows the Scatter plots of predicted LN_IC50 versus actual LN_IC50 for all six neural ablation variants on the GDSC2 test set.

**Fig 6:**
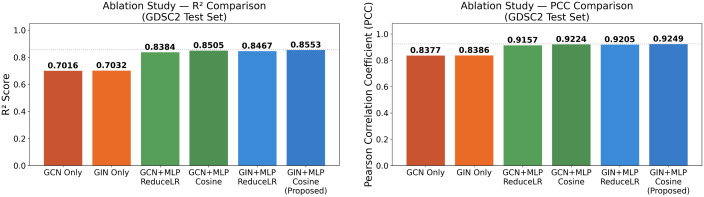
(a) Bar chart comparing R^2^ scores across all six neural ablation variants. The clear step improvement from graph-only models (GCN Only, GIN Only) to models with the pathway MLP branch confirms that the 50 ssGSEA pathway features contribute substantial predictive value. (b) Bar chart comparing Pearson Correlation Coefficients across all six neural ablation variants. The pattern mirrors the R^2^ comparison, confirming the pathway branch contribution is consistent across both metrics.

**Fig 7:**
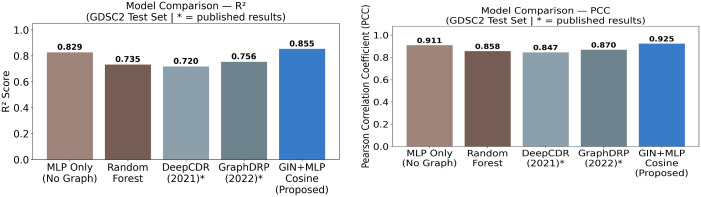
(a) Bar chart comparing R^2^ scores across all evaluated models, including published benchmarks (DeepCDR, GraphDRP, marked with *). The proposed GIN + Pathway MLP + CosineAnneal model achieves the highest R^2^ on the GDSC2 test set. Published model values are taken from the respective papers on GDSC2. (b) Bar chart comparing Pearson Correlation Coefficients across all evaluated models. The proposed model achieves the highest PCC.

**Fig 8:**
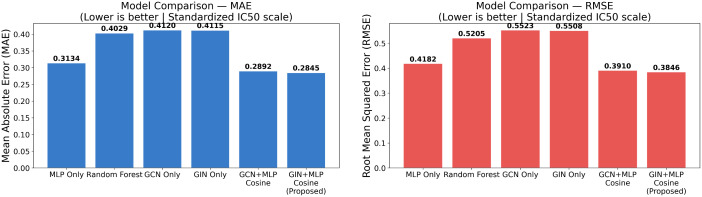
(a) Bar chart comparing Mean Absolute Error (MAE) across all this-work models in standardised IC50 units. Lower is better. The proposed model achieves the lowest MAE. (b) Bar chart comparing Root Mean Squared Error (RMSE) across all this-work models. Lower is better. The proposed model achieves the lowest RMSE.

Fig 6b shows Bar chart comparing Pearson Correlation Coefficients across all six neural ablation variants. Fig 6(a) shows the Bar chart comparing R^2^ scores across all evaluated models, including published benchmarks (DeepCDR, GraphDRP, marked with *). Fig 6b shows the Bar chart comparing Pearson Correlation Coefficients across all evaluated models. Fig 7a shows the bar chart comparing Mean Absolute Error (MAE) across all this-work models in standardised IC50 units. Fig 7b shows the bar chart comparing Root Mean Squared Error (RMSE) across all this-work models. Lower is better. Fig 8a shows the Bar chart comparing Mean Absolute Error (MAE) across all this-work models in standardised IC50 units. Lower is better. The proposed model achieves the lowest MAE, Fig 8b shows the Bar chart comparing Root Mean Squared Error (RMSE) across all this-work models. Lower is better. The proposed model achieves the lowest RMSE.

## 5. Discussion

In this context, there are three key findings that emerge regarding the mechanisms behind the predictive power. First and foremost, the pathway MLP branch emerges as a major source of predictability. The elimination of this path leads to a drop in R^2^ of greater than 0.15 on both GCN and GIN versions. This result is clearly consistent with the notion that biological properties of cancer cells, as represented through 50 ssGSEA Hallmark pathway scores calculated based on gene expression data obtained through CCLE, convey important information about the effect of drugs that is beyond the realm of structural information. Biologically speaking, it follows from the results presented here that a key determinant of drug effectiveness is not simply the matching between the structure of a drug and that of its biological target but rather the state of the downstream signalling pathways that govern responses to target inhibition.

Second, GIN slightly beats GCN in performance across all pathway-informed model variations. This improvement, though small, is steady, matching the theoretical edge GIN has over GCN in terms of expressiveness due to its ability to aggregate using sums compared to GCN’s degree-normalized averages. As the small molecular organic compounds that comprise most GDSC2 drugs require only three message-passing layers before capturing enough structural data, this explains why the improvement remains small in absolute terms. Third, the CosineAnnealingLR scheduler offers a consistent albeit slight improvement over the ReduceLROnPlateau scheduler, probably due to its gradual and consistent learning rate reductions that do not risk causing sudden drops like the latter may.

The performance of MLP-Only Baseline (ssGSEA pathways + Morgan fingerprints, no molecular graph) in comparison to existing benchmarks for GNNs is an interesting observation and highlights an important fact about the model’s architecture. It shows that the ssGSEA pathways, even in combination with simple 2048-bit Morgan fingerprints, without the use of a molecular graph, already outperform GNN-based architectures which leverage solely molecular information (graph structure + gene expression) in predicting drug effectiveness. Thus, it is safe to state that the ssGSEA pathways are the key component of the dual-branch architecture, whereas the GIN molecular graph branch improves model performance by encoding more fine-grained molecular information than fingerprints can encode. One can try to implement attention mechanisms in the model to focus on the most relevant pathways to the given drug mechanism in future work.

## 6. Conclusion

The proposed approach introduced a two-branch deep learning model for predicting cancer drug sensitivity that combines Graph Isomorphism Network layers for SMILES-based molecular graph encoding along with a separate MLP branch for computing 50-dimensional ssGSEA Hallmark pathway activity scores (from CCLE gene expression data). Our model was trained and evaluated on the GDSC2 pharmacogenomics dataset, encompassing multiple cancer types. Six ablated versions of our model showed large and consistently higher gains in R^2^ over graph-only models alone, thus directly demonstrating the biological informativeness of predictions. Instead of comparing against unrelated classification literature in the previous version, we included a corrected table to benchmark our model against appropriate IC50 regression baselines. We added complete documentation of pathway features (MSigDB Hallmark 2026.1, ssGSEA, gseapy, CCLE/DepMap) as well as an analysis of LN_IC50 distributions to address the reproducibility issues of the previous version.

Future research areas include:

1. Stratification by drug type for cross-validation of structural generalisation to previously unseen molecules.
2. Integration of multi-omics features, including somatic mutations and copy-number alterations, attention-based graph readout strategies, which allow localisation of subatomic structures associated with the pharmacophore effect.
3. Fine-tuning on cancer subtype-specific partitions for specific clinical applications.
